# Tribulations of a Traveller

**Published:** 1870-04

**Authors:** 


### TRIBULATIONS OF A TRAVELLER.

A New Yorker went to Germany with his family for purposes of education, intending to remain several years, and, as customary, rented a flat, or a whole story of a building, furnished nicely with all the conveniences of housekeeping. With suitable surroundings and fortunate concomitants this is the most comfortable, home-like, and economical method, escaping alike the bustle of the hotel and the want of privacy of a boarding-house. A lady correspondent writes, under date of March last, "Our New York friend has had great trouble with his landlady and servants, who clubbed together, and appropriated his wood and coal almost as fast as he could buy it. He laid in enough, as he thought, to last the whole winter, but in a few weeks he found he was entirely out. He laid in a new supply, and found the same thing happened again. At last he found them out; the servant told on the landlady and the landlady on the servant, who was dismissed, while the landlady agreed to abate from the rent. The servant begged pardon and was taken back, but she domanded to be supplied with beer; that was too much, so he ordered her off; she refused to go; he sent for a policeman who remained with the girl until she packed up her trunk, and then turned her out; she then sued for her wages for the whole term of her engagement, and that is what all strangers go through, more or less, who undertake housekeeping."

These are but a portion of the tribulations of travellers; at the same time, every one who can ought to go abroad, even if but for a few months. It improves the mind, invigorates the body, and gives a breadth of view which none can have who remain at home all their lives. Certainly great facilities of education are enjoyed in Germany at one quarter the rate in America, with more finished teachers. After living awhile in the country, and becoming better acquainted with the ways of the people, it becomes very pleasant. Still, for ourselves, we want no better place to live in than New York city, and we are never out of it forty-eight hours that we don't long to get back to its business, its bustle, its quiet and retirement, for when you get into your own house and the door is shut, you are just as quiet and retired as if in the recesses of the densest forests of the illimitable West. And then there is the morning paper and the "Evening Mail," the cozy fire, the bright gas lights, the every "modern convenience." Then, for evening amusements, there are lectures, and concerts, and prayer-meetings, and neighborly calls. If no one comes in, there are papers and the magazines, the tune-book and the piano, and that perfect " abandon " which a man has in his own home. If you get tired of the house, there is the clean sidewalk, upon which you can walk for miles, with variety in every block and a new face at every step.

But summer is coming, and almost every one wants to get out of the city, at least for a few weeks, and no class of persons need this recreation and change more than our hard-worked clergymen, and right glad are we to have learned that half fare only will be charged to clergymen and their families who want to make a visit by rail to San Francisco. What a glorious trip this would be to a clergyman; how it would unfold to him the extent and the grandeur of this country; what food for thought, for illustration. Reader! the very instant you finish this article, go and get two or three of the best men and women in your church, put your heads together, and concert some plan by which you may be able to say to your minister, Here is a ticket for a three months' absence and a passage by rail from here to San Francisco and back. Why, if you should do such a thing as this, you will feel the better for it, and the happier, all your days.

